# 基于超高效液相色谱-四极杆-飞行时间质谱的玉屏风颗粒化学成分及入血成分分析

**DOI:** 10.3724/SP.J.1123.2025.11008

**Published:** 2026-08-08

**Authors:** Wensen LU, Guanlin XIAO, Minshan WU, Jiaying WU, Quan LIU, Aili XU

**Affiliations:** 1. 广州中医药大学第五临床医学院，广东 广州 510405; 1. The School of the Fifth Clinical Medicine，Guangzhou University of Chinese Medicine，Guangzhou 510405，China; 2. 广东省中医药工程技术研究院，广东省中医药研究开发重点实验室，广东 广州 510095; 2. Guangdong Province Engineering Technology Research Institute of Traditional Chinese Medicine，Guangdong Provincial Key Laboratory of Research and Development in Traditional Chinese Medicine，Guangzhou 510095，China

**Keywords:** 玉屏风颗粒, 超高效液相色谱-四极杆-飞行时间质谱, 化学成分, 入血成分, Yupingfeng Granules, ultra performance liquid chromatography-quadrupole-time-of-flight mass spectrometry （UPLC-Q-TOF MS）, chemical constituents, bioavailable components

## Abstract

玉屏风颗粒作为中医经典名方，在免疫调节方面具有广泛应用，然而其复杂的化学组成及体内发挥药效的具体物质基础尚未完全阐明，尤其在病理状态下的入血成分研究相对缺乏。本研究旨在系统鉴定玉屏风颗粒的化学物质组，并进一步探究其在免疫缺陷模型大鼠体内的入血成分，以揭示其潜在的药效物质，为深入阐释该制剂的作用机制提供科学依据。研究采用超高效液相色谱-四极杆-飞行时间质谱（UPLC-Q-TOF MS）技术对玉屏风颗粒的化学成分及免疫缺陷模型大鼠给药后的入血成分进行分析。制剂和血清样本经处理，采用 Waters CORTECS UPLC C_18_色谱柱（150 mm×2.1 mm，1.6 μm），以0.1%甲酸水溶液-乙腈为流动相梯度洗脱，流速0.3 mL/min，柱温35 ℃，进样量3 µL，在电喷雾离子源正、负离子模式下采集数据后，通过与对照品保留时间、精确相对分子质量、二级碎片离子进行比对，并参照相关文献，完成化学成分的鉴定。从玉屏风颗粒中共鉴定出52种化学成分，包括11个黄酮类、13个有机酸类、13个苯丙素类、12个萜类成分、3个其他成分（醛类、核苷、氨基酸）。在此基础上，从给药大鼠的血清中鉴定出11个入血成分 。该研究在免疫缺陷病理模型条件下系统分析了玉屏风颗粒的入血成分，不仅明确了其体外化学物质基础，更进一步筛选出潜在的体内直接作用物质，为后续开展药效关联研究及作用机制探索提供了关键线索和依据

## Abstract (en)

Yupingfeng Granules， a classical and widely prescribed formula in traditional Chinese medicine， is extensively employed in clinical practice for its well-documented immunomodulatory and preventive activities. However， the highly complex chemical composition of this multi-herb formula and the specific material basis responsible for its therapeutic effects in vivo remain inadequately characterized and systematically elucidated. Research on its pharmacokinetically critical blood-entering components， which are indicative of systemic exposure and potential direct activity， particularly under representative pathological conditions， remains notably scarce and insufficient. This study therefore aims to systematically characterize the comprehensive chemical profile of Yupingfeng Granules by employing advanced analytical techniques such as HPLC-MS. Furthermore， it seeks to comprehensively investigate and characterize its blood-entering components specifically in cyclophosphamide-induced immunodeficient rat models， comparing them with normal physiological conditions where applicable. The overarching objective is to identify， quantify， and shed light on the formula′s potential active constituents that reach the systemic circulation. This work is expected to provide a more solid and detailed scientific basis for a deeper， molecular-level understanding of the pharmacodynamic mechanism of this important traditional Chinese medicine preparation and to inform its future rational clinical application and quality control. An ultra performance liquid chromatography-quadrupole-time-of-flight mass spectrometry （UPLC-Q-TOF MS） technology was used to identify the chemical composition of Yupingfeng Granules and the plasma-entering components after administration to immunodeficient model rats. The prepared granule and serum samples were analyzed using a Waters CORTECS UPLC C18 column （150 mm×2.1 mm， 1.6 μm） with gradient elution of 0.1% formic acid aqueous solution and acetonitrile as the mobile phases. The flow rate was set at 0.3 mL/min， the column temperature was maintained at 35 ℃， and the injection volume was 3 µL. Data were acquired in both positive and negative ion modes using an electrospray ionization source. The identification of chemical constituents was performed by comparing retention times， accurate relative molecular masses， and MS/MS fragment ions with those of reference standards， along with reference to relevant literature. Through a systematic analysis involving comparison of retention times， accurate molecular weights， and MS/MS fragment ions， combined with cross-referencing against relevant literature and authenticated reference standards， the chemical profile of Yupingfeng Granules and the bioactive components absorbed in vivo were comprehensively characterized. The investigation successfully identified a total of 52 chemical constituents in Yupingfeng Granules. Furthermore， 11 bioavailable components were detected in rat plasma after administration. These compounds encompass a diverse range of chemical classes， specifically including 11 flavonoids， 13 organic acids， 13 phenylpropanoids， 12 terpenoids， and 3 additional components categorized as aldehydes， nucleosides， and amino acids. This detailed identification provides a thorough overview of the chemical composition of the granules and elucidates the key constituents that are systemically absorbed. This study systematically analyzed the blood-entering components of Yupingfeng Granules under immunodeficient pathological conditions. This distinction is critically important， as the altered internal environment in a disease model may significantly influence the absorption， metabolism， and distribution of herbal compounds. The research not only clarifies the comprehensive chemical material basis of the formula in vitro by establishing a detailed chromatographic fingerprint but also， more significantly， bridges the gap to in vivo pharmacology by identifying which specific compounds from the complex mixture actually enter the systemic circulation. By correlating the in vitro chemical profile with the in vivo exposure data， the study effectively pinpoints a refined set of potential direct-acting substances that are most likely to contribute to the observed therapeutic effects. Consequently， these findings provide crucial experimental clues and a reliable analytical foundation for subsequent， more targeted research. This includes in-depth investigations into efficacy correlation—linking specific blood components to immunomodulatory activities—and the detailed exploration of its molecular mechanisms of action， thereby significantly advancing the scientific understanding of this classic formula.

玉屏风颗粒由黄芪、炒白术、防风三味中药组成，源于元代朱丹溪《丹溪心法》，具有扶正固本、益气补中的功效，临床上广泛应用于机体免疫力的提高以及免疫功能失衡引起的反复感冒^［1］^、过敏性鼻炎^［2］^、哮喘^［3］^等疾病。尽管玉屏风颗粒的临床应用历史悠久，但其药效物质尚不完全明确。《中国药典》^［4］^收载的玉屏风颗粒质量标准仅控制了黄芪甲苷的含量，但中药具有多成分、多靶点的特点，单一成分难以全面体现中药复方配伍的科学性和临床价值，而在中药复方的众多成分当中，只有能够最终进入体内的成分才是潜在的药效成分，因此，研究复方在体内的入血成分，能够大大缩小有效成分的筛选范围，是明确药效物质基础的前提条件。

中药血清药物化学是通过现代分析技术，鉴定含药血清中的移行成分，以阐明中药的药效物质基础及其体内过程的一门学科^［5］^。该学科通过对比给药血清与体外成分的差异，识别入血的原型成分及代谢产物，进而确定真正起效的物质。目前文献大多研究的是正常机体状态下的入血成分，但药物在体内的吸收利用与机体状态密切相关，其在病理状态下往往与正常生理状态存在显著差异^［6］^，因此，采用疾病模型动物来研究药物在病理状态下的入血成分，更能有效揭示中药的药效物质基础^［7］^。

本研究采用超高效液相色谱-四极杆-飞行时间质谱（UPLC-Q-TOF MS）技术，在免疫缺陷模型大鼠中进行玉屏风颗粒的入血成分鉴定。更符合玉屏风颗粒临床用于“免疫功能失衡”相关疾病的实际应用背景，提高了研究结果的临床相关性与药效物质基础的可靠性，为该制剂的质控提升和临床应用奠定基础。

## 1 实验部分

### 1.1 仪器、试剂与材料

ExionLC AC 型液相色谱X500R 质谱仪（美国Sciex公司）；Sorvall Legend Micro 17R型微量离心机（美国Thermo Scientific公司）；XS205型电子分析天平（瑞士Mettler-Toledo公司）。

玉屏风颗粒（批号：241102，国药集团广东环球制药有限公司）。绿原酸（批号：110753-202119，纯度 96.2%）、亥茅酚（批号：735-46-6）、黄芪甲苷（批号：84680-75-1）、黄芪皂苷Ⅲ（批号：H-036-160908）、异阿魏酸（批号：111698-201904）、水杨酸（批号：100106-202106）、壬二酸（批号：101067-200901）对照品购自中国食品药品检定研究院。夏佛塔苷（批号：RS07761120）、毛蕊异黄酮葡萄糖苷（批号：RS08821120）、白术内酯Ⅱ（批号：RS07061120）、白术内酯Ⅲ（批号：RS07081120）、染料木苷（批号：RS02431120）对照品购自上海诗丹德标准技术服务有限公司。异绿原酸 B（批号：RDD-Y 06911812012，纯度 98%）、隐绿原酸（批号：AF9080321，纯度98%）、新绿原酸（批号：RDD-X01402112013，纯度98%）、对羟基苯甲酸（批号：D-063-161216）、对羟基苯甲醛（批号：D-058-181216）、花椒毒素（批号：H-035-170323）对照品购自成都瑞芬思生物科技有限公司。异绿原酸A（批号：RDD-Y06911812012，纯度98%）对照品购自成都瑞芬思德丹生物科技有限公司。升麻素（批号：20120511）、升麻素苷（批号：101617）、5-*O*-甲基维斯阿米醇（批号：101662）、5-*O*-甲基维斯阿米醇苷（批号：YJ-100829）、芒柄花素（批号：100390）、白术内酯Ⅰ（批号：YJ0282）、亥茅酚苷（批号：103613）对照品购自江苏永健医药科技有限公司。未标明纯度的对照品纯度均大于 98%。实验用水为娃哈哈纯净水，甲醇、乙腈、甲酸均为质谱纯（美国 Fisher Chemical公司），其余试剂均为分析纯。

SPF级雄性SD大鼠24只，体质量160~180 g，日龄45~50 d，购自广东省医学实验动物中心。动物生产许可证号：SCXK（粤）2022-0002。实验前适应性喂养1周，自由饮水、饮食。饲养条件：温度23~26 ℃，相对湿度45%~55%，12 h/12 h昼夜交替。本实验过程对动物处理均符合广东省第二中医院（广东省中医药工程技术研究院）实验动物伦理委员会相关规定（伦理批准编号：049382）。

### 1.2 对照品溶液的制备

分别精密称取各对照品适量，置于10 mL容量瓶中，加甲醇超声溶解并定容至刻度，制成各对照品质量浓度均为40 µg/mL的混合对照品溶液，用0.22 µm微孔滤膜滤过，即得。

### 1.3 供试品溶液的制备

取玉屏风颗粒1 g，置于25 mL量瓶中，加入50%乙醇水溶液定容至刻度，超声处理30 min，4 ℃、12 000 r/min离心10 min，收集上清液，0.22 μm微孔滤膜滤过，即得。

### 1.4 动物分组与造模

24只SPF级雄性SD大鼠，随机均分成3组，分为空白对照组、模型组、给药组。空白组、模型组6只，给药组12只。适应性喂养1周后，参考文献［^8^，^9^］方法建立大鼠免疫缺陷模型，具体如下：除空白对照组外，其余各组于实验1~7 d，按40 mg/kg腹腔注射环磷酰胺，首次加倍，隔日一次，连续4次，正常组腹腔注射等剂量的生理盐水。

### 1.5 给药与血样采集

实验前禁食12 h，不禁水，给药组灌胃给予玉屏风颗粒提取物水液（5.4 g生药/kg），空白组和模型组灌胃给予蒸馏水。末次给药后，于0、5、30、60、90、120、180 min 眼眶取血各约0.5 mL，静置30 min，4 ℃、4 000 r/min 离心15 min，取上清， -80 ℃保存。空白血清收集方式同含药血清。

### 1.6 血清样品的处理

取给药组各时间点的血清等比例混合，取其中600 μL加入预冷的甲醇2.4 mL，涡旋混匀30 s，-20 ℃放置1 h，4 ℃、12 000 r/min离心15 min，取全部上清液至离心管内，氮气吹干，加入150 μL预冷的甲醇复溶，涡旋30 s，4 ℃、12 000 r/min离心15 min，吸取上清液90 μL置于进样小瓶。

### 1.7 色谱和质谱条件

色谱柱：Waters CORTECS UPLC C_18_色谱柱（150 mm×2.1 mm，1.6 μm）；柱温：35 ℃；进样量：3 µL；流速：0.3 mL/min；流动相A：0.1%甲酸水溶液，流动相B：乙腈。梯度洗脱程序：0~4 min，5%B~20%B；4~15 min，20%B~40%B；15~20 min，40%B~45%B；20~24 min，45%B~55%B；24~27 min，55%B~80%B；27~31 min，95%B；31~34 min，95%B；34~35 min，95%B~5%B；35~40 min，5%B。

电喷雾离子源（ESI）；正离子和负离子模式同时扫描；喷雾电压：5 500 V（ESI^+^）/4 500 V（ESI^-^），裂解电压：100 V（ESI^+^）/80 V（ESI^-^），毛细管电压：5 500 V（ESI^+^）/4 500 V（ESI^-^），碰撞能量：35 eV（ESI^+^）/-35 eV（ESI^-^），质量扫描范围：*m/z* 100~1 000，离子源温度：500 ℃，气帘气压力：0.24 MPa，雾化气与辅助气压力：0.37 MPa。

### 1.8 化学成分鉴定及入血成分确定

通过检索CNKI、SciFinder、ChemicalBook、PubChem及TCMSP等数据库，系统搜集与整合玉屏风颗粒所含的化学成分信息，并据此建立本地化合物数据库。利用SCIEX OS 3.1.0软件进行色谱峰的提取与匹配分析，设定质量偏差不超过0.000 5%、同位素丰度偏差在5%以内、数据库匹配分数大于等于9的标准，将所得数据与数据库中化合物的准分子离子峰和碎片裂解模式进行比对，从而准确鉴定玉屏风颗粒及血清样品中的化学成分。将给药前（0 h）和给药后的血清谱图、玉屏风颗粒的谱图进行比较，扣除血清中的内源性成分，确定玉屏风颗粒的入血成分。

## 2 结果与讨论

### 2.1 玉屏风颗粒成分鉴定

在正、负离子模式下，获得玉屏风颗粒、空白血清和含药血清的总离子流色谱图，如图1所示。对供试品溶液的保留时间、质谱碎片等信息进行分析，确定玉屏风颗粒中存在的成分。在正、负离子模式下共鉴定出化学成分52个，包括12个黄酮类、13个有机酸类、13个苯丙素类、12个萜类成分、3个其他成分（醛类、核苷、氨基酸）。25个成分经对照品比对，详见表1。

**图1:**
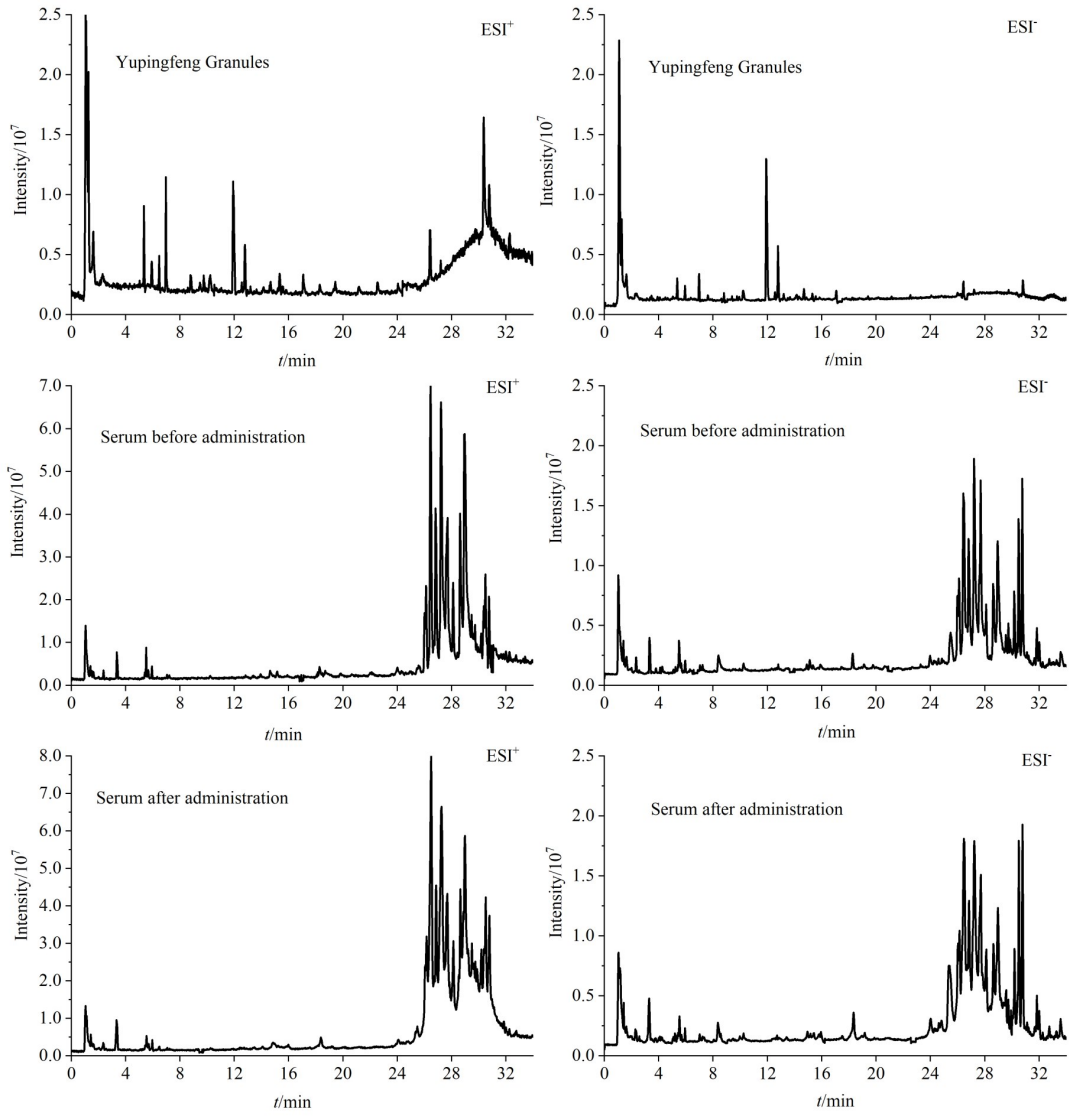
玉屏风颗粒和给药前、后血清样品在正、负离子模式下的总离子流色谱图

**表1:** 玉屏风颗粒的化学成分鉴定结果

No.	*t*_R_/min	Measured（*m/z*）	Theoretical（*m/z*）	Mass error/10^-6^	Measured MS^2^ （*m/z*）	Quasimolecular ion	Compound	Molecular formula	Compound classification	Origin plant
1	1.1877	133.0139	133.0142	-2.3	71.0133，115.0031	［M-H］^-^	malic acid	C_4_H_6_O_5_	organic acids	H
2	1.2613	268.1040	268.1040	0	136.0613	［M+H］^+^	adenosine	C_10_H_13_N_5_O_4_	nucleoside	H
3	1.2632	191.0193	191.0197	-2.1	87.0082，111.0079，147.0283	［M-H］^-^	citric acid	C_6_H_8_O_7_	organic acids	H
4	2.3009	191.0559	191.0561	-1.0	72.9926，101.0604，129.0561	［M-H］^-^	quinic acid	C_7_H_12_O_6_	organic acids	H
5	2.3233	164.0717	164.0717	0	103.0546，147.0444	［M-H］^-^	phenylalanine	C_9_H_11_NO_2_	amino acids	H
6	3.0859	353.0877	353.0878	-0.2	135.0451，179.0347，191.0561	［M-H］^-^	neochlorogenic acid^*^	C_16_H_18_O_9_	phenylpropanoids	F
7	3.7984	137.0242	137.0244	-1.8	65.0397，92.0255，	［M-H］^-^	4-hydroxybenzoic acid ^*^	C_7_H_6_O_3_	organic acids	F
8	3.8730	353.0879	353.0878	0.4	111.0458，173.0455，233.0471	［M+H］^-^	chlorogenic acid^*^	C_16_H_18_O_9_	phenylpropanoids	F
9	4.0581	353.0879	353.0878	0.4	93.0343，135.0446，173.0445	［M-H］^-^	cryptochlorogenic acid ^*^	C_16_H_18_O_9_	phenylpropanoids	F
10	4.9179	121.0294	121.0295	-0.9	65.0384，92.0262，121.0295	［M-H］^-^	4-hydroxybenzaldehyde ^*^	C_7_H_6_O_2_	aldehydes	F
11	5.0602	461.1100	461.1089	2.3	135.0088，284.0329，299.0564	［M-H］^-^	calycosin-7-*O*-*β*-D-glucopyranoside	C_22_H_22_O_11_	flavonoids	H
12	5.1408	565.1556	565.1552	0.8	349.0695，379.0826，409.0933	［M+H］^+^	schaftoside^*^	C_26_H_28_O_14_	flavonoids	H
13	5.3432	469.1684	469.1704	-4.3	189.0550，261.1117，290.1146，307.1161	［M+H］^+^	cimicifugoside^*^	C_22_H_28_O_11_	phenylpropanoids	F
14	5.5692	163.0400	163.0401	-0.7	93.0346，119.0499	［M-H］^-^	*cis*-4-coumaric acid	C_9_H_8_O_3_	organic acids	F
15	5.9047	447.1276	447.1286	-2.2	137.0238，253.0500，270.2520，285.0744	［M+HCOO］^+^	calycosin-7-*O*-*β*-D-glucoside^*^	C_22_H_22_O_10_	flavonoids	H
16	6.1349	193.0506	193.0506	0	134.0294，149.0644，178.0284	［M-H］^-^	ferulic acid^*^	C_10_H_10_O_4_	organic acids	F
17	6.3221	193.0506	193.0506	0	134.0373	［M-H］^-^	isoferulic acid^*^	C_10_H_10_O_4_	organic acids	F
18	6.4586	307.1163	307.1176	-4.4	207.0657，235.0451，259.0597，289.1072	［M+H］^+^	cimifugin^*^	C_16_H_18_O_6_	phenylpropanoids	F
19	6.6573	515.1204	515.1195	1.8	173.0448，353.0913	［M-H］^-^	isochlorogenic acid B^*^	C_25_H_24_O_12_	phenylpropanoids	F
20	6.9108	515.1212	515.1195	3.4	173.0448，353.0913	［M-H］^-^	isochlorogenic acid A^*^	C_25_H_24_O_12_	phenylpropanoids	F
21	6.9616	453.1736	453.1755	-4.3	231.0645，273.1110，291.1213	［M+H］^+^	5-*O*-methylvisammioside^*^	C_22_H_28_O_10_	phenylpropanoids	F
22	7.4752	515.1203	515.1195	1.6	173.0458，353.0884	［M-H］^-^	isochlorogenic acid C^*^	C_25_H_24_O_12_	phenylpropanoids	F
23	7.5913	187.0969	187.0976	-3.5	69.0351，97.0656，125.0965，143.1081，169.0861	［M-H］^-^	azelaic acid^*^	C_9_H_16_O_4_	organic acids	B
24	7.7207	137.0242	137.0244	-1.3	65.0392，93.0340	［M-H］^-^	salicylic acid^*^	C_7_H_6_O_3_	organic acids	F
25	8.7877	431.1330	431.1337	-1.5	269.0797	［M+H］^+^	ononin^*^	C_22_H_22_O_9_	flavonoids	H
		429.1239	429.1246	-1.3	252.0431，267.0652	［M-H］^-^		C_22_H_22_O_9_		
26	9.1671	253.0508	253.0506	0.8	133.0307	［M-H］-	daidzein^*^	C_15_H_10_O_4_	flavonoids	H
27	9.4936	291.1221	291.1227	-2.0	191.0709，205.0501，243.0654	［M+H］^+^	5-*O*-methylvisamminol^*^	C_16_H_18_O_5_	phenylpropanoids	F
28	9.7565	301.1070	301.1071	-0.3	106.0420，152.0477，167.0708	［M+H］^+^	methylnissolin	C_17_H_16_O_5_	flavonoids	H
		299.0929	299.0925	1.3	95.0128，213.0557，284.0695，269.0460	［M-H］^-^		C_17_H_16_O_5_		
29	10.1593	285.0751	285.0757	-2.4	137.0237，213.0548，270.0521	［M+H］^+^	calycosin	C_16_H_12_O_5_	flavonoids	H
		283.0605	283.0612	-2.4	135.0081，211.0398	［M-H］^-^		C_16_H_12_O_5_		
30	10.2598	439.1597	439.1599	-0.3	217.0502，259.0969，277.1067	［M+H］^+^	*sec*-*O*-glucosylhamaudol^*^	C_21_H_26_O_10_	phenylpropanoids	F
		437.1453	437.1453	1.4	89.0254，152.9924，257.0849	［M-H］^-^		C_21_H_26_O_10_		
31	10.2659	463.1611	463.1610	0.3	286.0857，303.1086	［M-H］^-^	isomucronulatol 7-*O*-*β*-glucoside	C_23_H_28_O_10_	flavonoids	H
32	11.9498	965.4222	965.4235	-1.4	803.3681	［M-H］^-^	rebaudioside A	C_44_H_70_O_23_	terpenoids	H
33	12.4319	217.0495	217.0495	0	146.0372，174.0313	［M+H］^+^	xanthotoxin^*^	C_12_H_8_O_4_	phenylpropanoids	F
34	12.5297	970.5073	970.5108	-3.6	970.5073	［M+Na］^+^	astragaloside Ⅶ	C_47_H_78_O_19_	terpenoids	H
		991.5142	991.5119	2.3	945.5112	［M+HCOO］^-^		C_47_H_78_O_19_		
35	13.8331	275.0928	275.0925	1.1	275.0928	［M-H］^-^	hamaudol^*^	C_15_H_16_O_5_	phenylpropanoids	F
		277.1071	277.1071	0	205.0500，259.0965	［M+H］^+^		C_15_H_16_O_5_		
36	14.0854	255.0666	255.0663	1.1	91.0195，119.0500，135.0099	［M-H］^-^	isoliquiritigenin	C_15_H_12_O_4_	flavonoids	H
37	14.6109	267.0658	267.0663	-1.7	195.0454，223.0403，252.0420	［M-H］^-^	formononetin^*^	C_16_H_12_O_4_	flavonoids	H
		269.0805	269.0808	-1.3	181.0653，197.0601，253.0499	［M+H］^+^		C_16_H_12_O_4_		
38	15.1842	299.0915	299.0914	0.5	211.0764，256.0747，284.0690，299.0922	［M+H］^+^	5-hydroxy-7，8-dimethoxyflavone	C_17_H_14_O_5_	flavonoids	H
39	15.4438	808.4538	808.4580	-5.2	808.4512	［M+Na］^+^	astragaloside A^*^	C_41_H_68_O_14_	terpenoids	H
		829.4583	829.4591	-1.0	783.4541	［M+HCOO］^-^		C_41_H_68_O_14_		
40	15.4438	808.4544	808.4580	-4.4	808.4544	［M+Na］^+^	astragaloside Ⅲ^*^	C_41_H_68_O_14_	terpenoids	H
		829.4583	829.4591	-1.0	783.4541	［M+HCOO］^-^		C_41_H_68_O_14_		
41	17.2110	850.4665	850.4685	-4.7	850.4665	［M+Na］^+^	astragaloside Ⅱ^*^	C_43_H_70_O_15_	terpenoids	H
		871.4692	871.4697	-0.6	765.4470，825.4681	［M+HCOO］^-^		C_43_H_70_O_15_		
42	17.9040	941.5117	941.5115	0.2	941.5142	［M-H］^-^	soyasaponin Bb	C_48_H_78_O_18_	terpenoids	H
43	19.3730	249.1485	249.1485	0	203.1441，231.1386	［M+H］^+^	atractylenolide Ⅲ^*^	C_15_H_20_O_3_	terpenoids	B
44	21.3641	892.4748	892.4791	-4.8	892.4748	［M+Na］^+^	astragaloside Ⅰ^*^	C_45_H_72_O_16_	terpenoids	H
		913.4800	913.4802	-0.3	867.4799	［M+HCOO］^-^		C_45_H_72_O_16_		
45	24.6322	233.1536	233.1536	0	91.0551，105.0703，131.0861	［M+H］^+^	atractylenolide Ⅱ^*^	C_15_H_20_O_2_	terpenoids	B
46	26.2772	934.4858	934.4896	-4.1	934.4858	［M+Na］^+^	acetytastragaloside	C_47_H_74_O_17_	terpenoids	H
		955.4921	955.4908	1.3	955.4958	［M+HCOO］^-^		C_47_H_74_O_17_		
47	27.0786	231.1381	231.1380	0.6	55.0540，94.0654，149.0895	［M+H］^+^	atractylenolide Ⅰ^*^	C_15_H_18_O_2_	terpenoids	B
48	29.4004	297.2414	297.2424	-3.5	297.2414	［M-H］^-^	13（*R*）- hydroxy-9Z，11E-octadecadienoic acid	C_18_H_32_O_3_	organic acids	B
49	29.7256	277.2171	277.2173	-0.6	59.0130，120.0210	［M-H］^-^	linolenic acid	C_18_H_30_O_2_	organic acids	B
		279.2319	279.2319	0	67.0544，81.0701	［M+H］^+^		C_18_H_30_O_2_		
50	30.7576	279.2324	279.2330	-1.8	59.0138，261.2232	［M-H］^-^	linoleic acid	C_18_H_32_O_2_	organic acids	B
		281.2476	281.2475	0.4	55.0544，69.0701，95.0859	［M+H］^+^		C_18_H_32_O_2_		
51	31.9920	281.2488	281.2486	0.7	165.1281	［M-H］^-^	oleic acid	C_18_H_34_O_2_	organic acids	B
52	32.5003	291.1743	219.1743	0	291.1743	［M+H］^+^	Selina-4（15），7（11）-dien-8-one	C_15_H_22_O	terpenoids	B

∗ Confirmed by standard substance； H： *Astragalus membranaceus* （Fisch.） Bunge.； B： *Atractylodes macrocephala* Koidz.； F： *Saposhnikovia divaricata* （Turcz.） Schischk.

### 2.2 玉屏风颗粒入血成分鉴定

将给药前、后血清谱图和玉屏风颗粒的谱图进行比较，并扣除血清中的内源性成分。分析发现血清中含有11个成分。包括黄酮类成分3个、苯丙素类5个、有机酸类1个、萜类2个，其中9个成分经对照品比对，详见表2。入血成分中以黄酮类和苯丙素类为主，6个来源于防风，5个来源于黄芪。入血成分鉴定数量相对较少且并无来自白术的入血成分，推测其原因可能是部分成分口服生物利用度低，在胃肠道被水解或菌群代谢，在肝脏发生首过效应等，导致浓度极低，未能达到检出限。入血成分中，升麻素、升麻素苷、5-*O*-甲基维斯阿米醇、5-*O*-甲基维斯阿米醇苷均为防风特征苯丙素类成分，文献［^10^］证实其具有显著的抗炎、镇痛、解热及抗过敏活性，这直接印证了防风在方中“祛风解表”的传统功效，可能是玉屏风颗粒用于治疗表虚自汗、易感风邪等证的关键物质之一。芒柄花素、黄芪甲苷，则是黄芪的标志性黄酮及皂苷类成分。文献［^11^］表明，这些成分具有强大的免疫调节、抗炎、抗氧化及增强机体屏障功能的作用，这与黄芪“益气固表”的核心功效高度契合，为玉屏风颗粒提升机体免疫力、巩固肌表提供了科学依据。

**表2:** 玉屏风颗粒的入血成分鉴定结果

No.	*t*_R_/min	Measured *（m/z）*	Theoretical *（m/z）*	Mass error/10^-6^	Measured MS （*m/z*）	Quasimolecular ion	Compound	Molecular formula	Compound classification	Origion plant
1	5.3432	469.1684	469.1704	-4.3	189.0550，261.1117，290.1146，307.1161	［M+H］^+^	cimicifugoside^*^	C_22_H_28_O_11_	phenylpropanoids	F
2	6.4586	307.1163	307.1176	-4.4	207.0657，235.0451，259.0597，289.1072	［M+H］^+^	cimifugin^*^	C_16_H_18_O_6_	phenylpropanoids	F
3	6.9616	453.1736	453.1755	-4.3	231.0645，273.1110，291.1213	［M+H］^+^	5-*O*-methylvisammioside^*^	C_22_H_28_O_10_	phenylpropanoids	F
4	7.7207	137.0242	137.0244	-1.3	65.0392，93.0340	［M-H］^-^	salicylic acid^*^	C_7_H_6_O_3_	organic acids	F
5	9.1671	253.0508	253.0506	0.8	133.0307	［M-H］^-^	daidzein^*^	C_15_H_10_O_4_	flavonoids	H
6	9.4936	291.1221	291.1227	-2.0	191.0709，205.0501，243.0654	［M+H］^+^	5-*O*-methylvisamminol^*^	C_16_H_18_O_5_	phenylpropanoids	F
7	9.7565	301.1070	301.1071	-0.3	106.0420，152.0477，167.0708	［M+H］^+^	methylnissolin	C_17_H_16_O_5_	flavonoids	H
		299.0929	299.0925	1.3	95.0128，213.0557，284.0695，269.0460	［M-H］^-^		C_17_H_16_O_5_		
8	11.9498	965.4222	965.4235	-1.4	803.3681	［M-H］^-^	rebaudioside A	C_44_H_70_O_23_	terpenoids	H
9	13.8331	275.0928	275.0925	1.1	275.0928	［M-H］^-^	hamaudol^*^	C_15_H_16_O_5_	phenylpropanoids	F
		277.1071	277.1071	0	205.0500，259.0965	［M+H］^+^		C_15_H_16_O_5_		
10	14.6109	267.0658	267.0663	-1.7	195.0454，223.0403，252.0420	［M-H］^-^	formononetin^*^	C_16_H_12_O_4_	flavonoids	H
		269.0805	269.0808	-1.3	181.0653，197.0601，253.0499	［M+H］^+^		C_16_H_12_O_4_		
11	15.4438	808.4538	808.4580	-5.2	808.4512	［M+Na］^+^	astragaloside A^*^	C_41_H_68_O_14_	terpenoids	H
		829.4583	829.4591	-1.0	783.4541	［M+HCOO］^-^		C_41_H_68_O_14_		

### 2.3 玉屏风颗粒成分质谱解析

#### 2.3.1 黄酮类化合物质谱解析

黄酮类化合物的基本结构为2-苯基色原酮，其分子中普遍具有C_6_-C_3_-C_6_的骨架类型，该类成分在自然界中既有以游离状态存在的，也常见与糖类结合成苷。在质谱分析中，主要的裂解方式为C环发生逆狄尔斯-阿德尔反应（RDA）裂解、糖苷键断裂、脱去中性碎片、脱水等碎片离子^［12］^。表1中化合物15在正离子模式下检测到准分子离子峰为*m/z* 447.127 6 ［M+H］^+^，推断其分子式为C_22_H_22_O_10_，二级质谱中主要的碎片离子如下：准分子离子脱去一分子葡萄糖后，生成*m/z* 285.074 4的［M+H-Glu］^+^，该离子进一步失去CH_3_，产生*m/z* 270.252 0 的［M+H-Glu-CH_3_］^+^。此外，通过RDA裂解得到碎片离子*m/z* 137.023 8，与文献［^13^］及对照品对比，推断该化合物为毛蕊异黄酮葡萄糖苷。可能的质谱裂解途径见图2a。表1中化合物25在负离子模式下的检测到准分子离子峰为*m/z* 429.123 9 ［M-H］^-^，在二级质谱图中产生*m/z* 267.065 2 ［M-H-C_6_H_10_O_5_］^-^和*m/z* 252.043 1 ［M-H-C_6_H_10_O_5_-CH_3_］^-^的碎片离子，通过质谱信息与文献［^14^］及对照品对比，推断化合物为芒柄花苷，可能的质谱裂解途径如图2b。

**图2:**
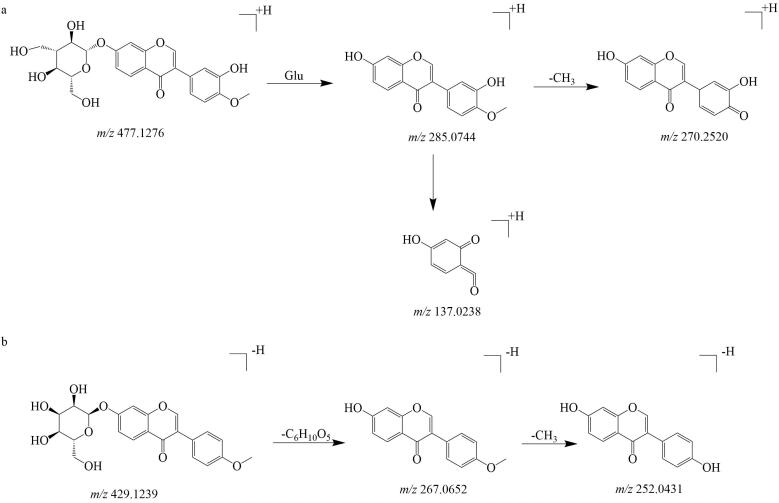
（a）毛蕊异黄酮葡萄糖苷和（b）芒柄花苷的质谱裂解途径

#### 2.3.2 苯丙素类化合物质谱解析

苯丙素类化合物是一类以C_6_-C_3_（1个苯环与3个直链碳原子）为基本结构片段的天然产物，其典型代表包括苯丙酸、香豆素和木质素等。在质谱分析中，该类化合物常表现出易于丢失CO、CO₂及H₂O等小分子中性碎片的特点。以表1中化合物18为例，其在正离子模式下的准分子离子为*m/z* 307.116 3［M+H］^+^，推断其分子式为C_16_H_18_O_6_，失去H_2_O后产生*m/z* 289.107 2的［M+H-H_2_O］^+^，再失去两分子CH_3_产生*m/z* 259.059 7的［M+H-H_2_O-2CH_3_］^+^；*m/z* 235.045 1的［M+H-C_4_H_8_O］^+^是由*m/z* 307.116 3的［M+H］^+^的呋喃环裂解产生，*m/z* 235.045 1的［M+H-C_4_H_8_O］^+^失去CO产生*m/z* 207.065 7的［M+H-C_4_H_8_O-CO］^+^，裂解规律与对照品基本一致，参考文献［^15^］推测其为升麻素，其可能的裂解途径见图3a。表1中化合物6在负离子模式下检测到准分子离子峰*m/z* 353.087 7 ［M-H］^-^，推测其分子式为C_16_H_18_O_9_。二级质谱分析显示，该离子可通过两种途径发生裂解：其一为丢失C_7_H_10_O_5_，产生*m/z* 179.034 7的［M-H-C_7_H_10_O_5_］^-^，再丢失CO_2_，产生*m/z* 135.045 1的［M-H-C_7_H_10_O_5_-CO_2_］^-^；其二为脱去C_9_H_6_O_3_，生成*m/z* 191.056 1的［M-H-C_9_H_6_O_3_］^-^，通过质谱信息与文献［^16^］及对照品对比，推断该化合物为新绿原酸，其可能的质谱裂解规律见图3b。

**图3:**
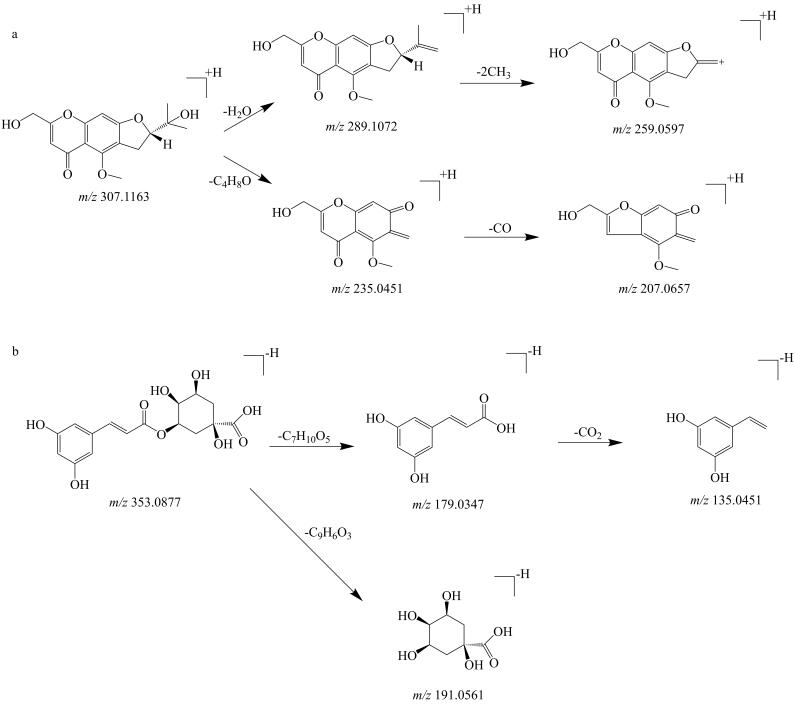
（a）升麻素和（b）新绿原酸的质谱裂解途径

#### 2.3.3 有机酸类化合物质谱解析

有机酸类成分易发生脱羧、脱甲基等反应。以表1中化合物16为例，其在负离子模式下的准分子离子为*m/z* 193.050 6［M-H］^-^，推断分子式为C_10_H_10_O_4_，丢失一分子甲基产生碎片*m/z* 178.028 4的［M-H-CH_3_］^-^，丢失CO_2_产生*m/z* 149.064 4的［M-H-CO_2_］^-^，在此基础上继续丢失CH_3_产生*m/z* 134.029 7的［M-H-CO_2_-CH_3_］^-^。结合文献［^17^］及对照品对比，推断该化合物为阿魏酸，可能的裂解途径见图4a。表1中化合物23在负离子模式下的准分子离子峰为*m/z* 187.096 9 ［M-H］^-^，其二级质谱呈现连续裂解过程：先失去H_2_O得到*m/z* 169.086 1的［M-H-H_2_O］^-^，或脱去CO_2_得到*m/z* 143. 108 1的［M-H-CO_2_］^-^，该离子进一步失去H_2_O得到*m/z* 125. 096 5的［M-H-CO_2_-H_2_O］^-^，随后脱去乙基单元C_2_H_4_获得*m/z* 97.065 6的［M-H-CO_2_-H_2_O-C_2_H_4_］^-^，并继续丢失另一分子 C_2_H_4_，最终产生*m/z* 69.035 1的［M-H-CO_2_-H_2_O-2C_2_H_4_］^-^，结合裂解规律及文献［^18^］报道，推断其为壬二酸，可能的质谱裂解行为见图4b。

**图4:**
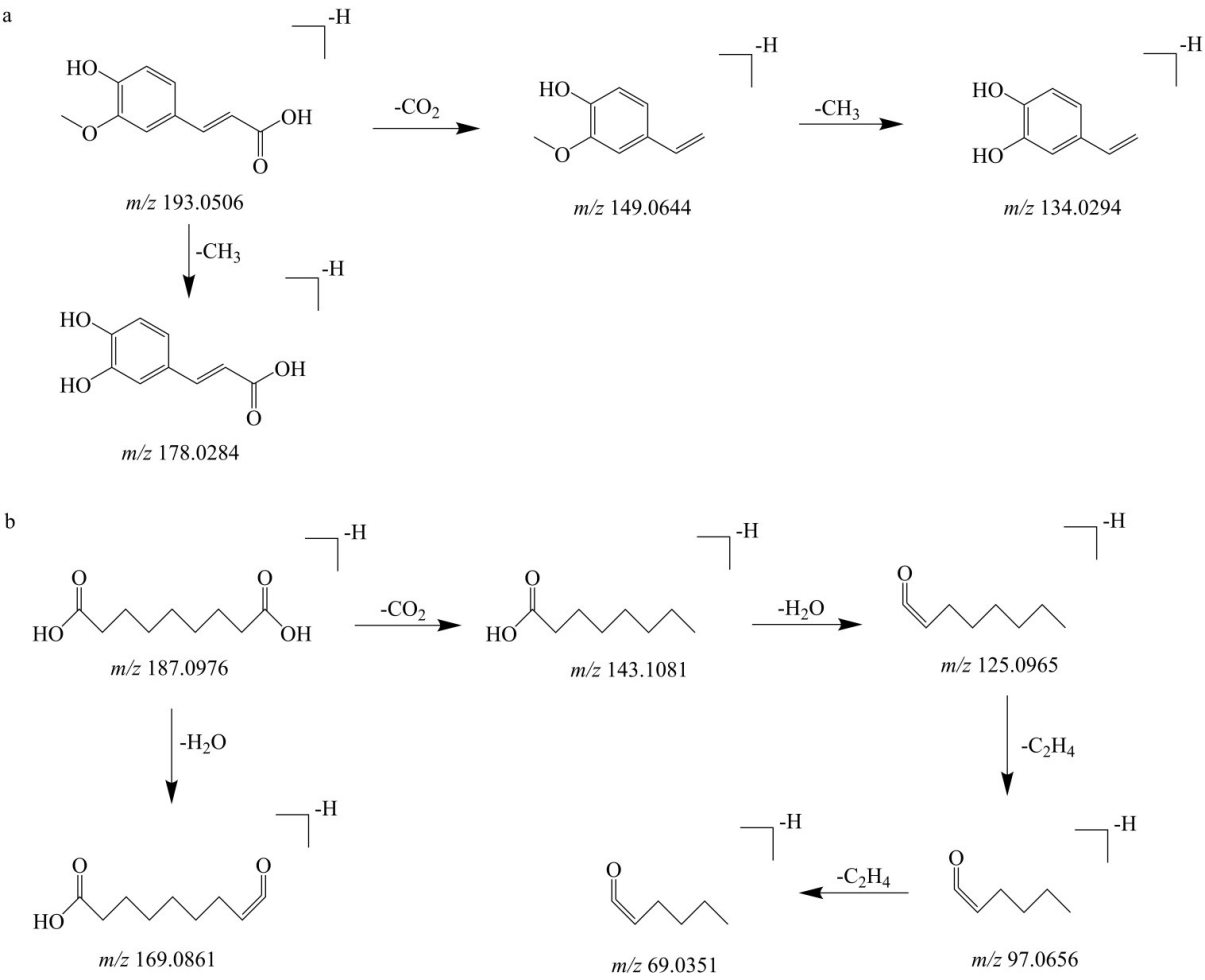
（a）阿魏酸和（b）壬二酸的质谱裂解途径

#### 2.3.4 萜类化合物质谱解析

作为一类广泛存在的重要天然产物，萜类化合物具有以异戊二烯（C₅）为通用结构单元的聚合体结构。其在质谱中响应良好，常见的裂解途径为脱掉连接的糖苷基、侧链部分易断裂-C *_x_* H *_y_* 或C *_x_* H *_y_* O小基团以及-OH等^［19］^。表1中化合物41在负离子模式下的准分子离子峰为*m/z* 871.469 7［M+HCOOH］^-^，推断分子式为C_43_H_70_O_15_。在二级质谱中，准分子离子丢失一分子甲酸，产生*m/z* 825.468 1的［M-H］^-^，该碎片脱去-C_2_H_4_O_2_产生*m/z* 765.447 0的［M-H-C_2_H_4_O_2_］^-^，通过质谱信息与文献［^20^］及对照品对比，推断该化合物为黄芪皂苷Ⅱ，可能的裂解途径见图5a，表1中化合物43在正离子模式下的准分子离子峰为*m/z* 249.148 5［M+H］^+^，推断分子式为C_15_H_20_O_3_，在二级质谱中，准分子离子丢失H_2_O形成*m/z* 231.138 6的［M+H-H_2_O］^+^，再失去CO形成*m/z* 203.144 1的［M+H-H_2_O-CO］^+^，经文献［^21^］及对照品对比，推断该化合物为白术内酯Ⅲ，可能的裂解途径见图5b。

**图5:**
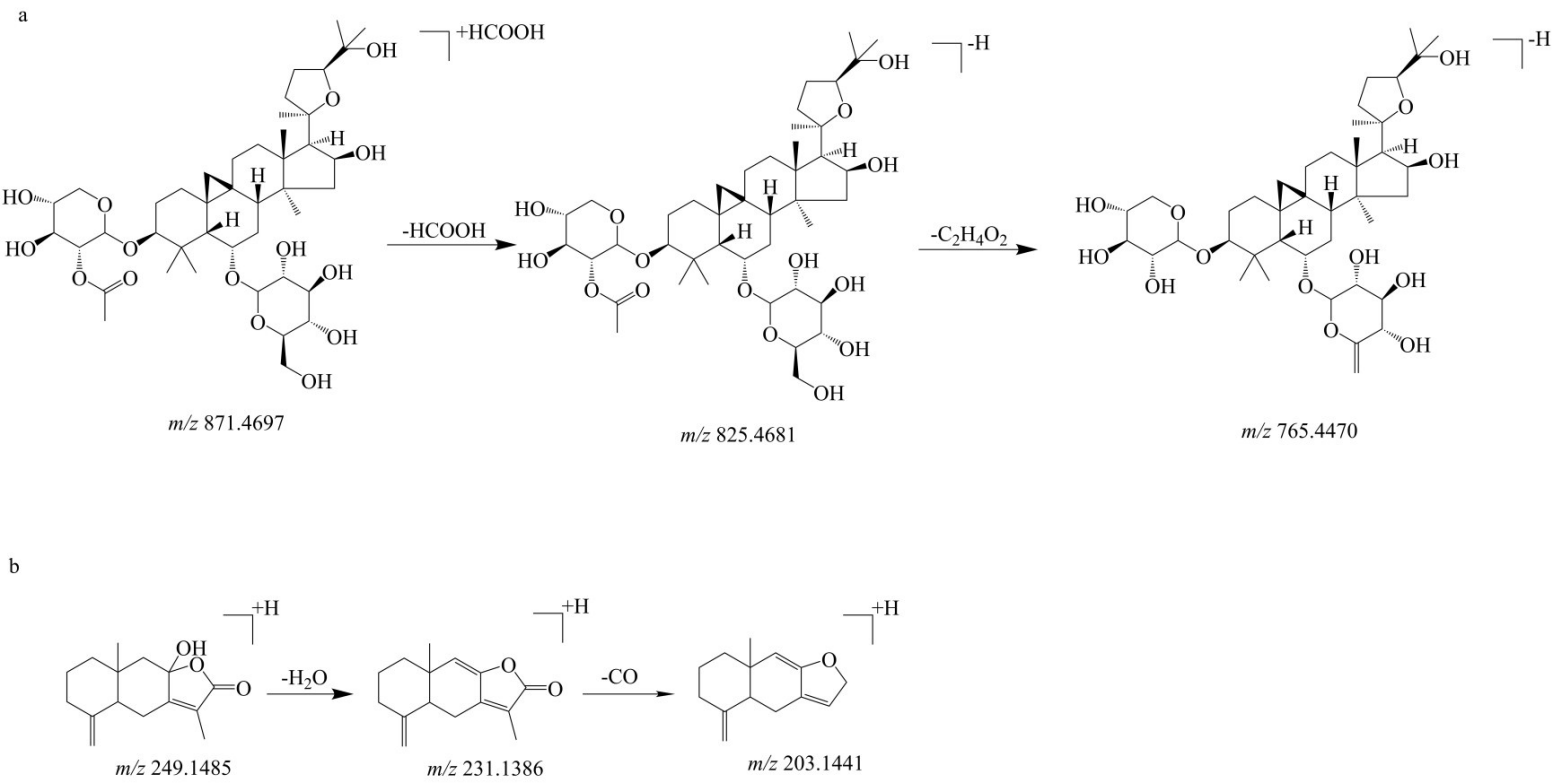
（a）黄芪皂苷Ⅱ和（b）白术内酯Ⅲ的质谱裂解途径

## 3 结论

本研究通过UHPLC-Q-TOF MS技术系统分析了玉屏风颗粒的化学成分与在免疫缺陷模型大鼠体内的入血成分，共鉴定出52个化合物，包括黄酮类、苯丙素类、有机酸类、萜类等多种结构类型，并进一步确认了11个原型入血成分。虽未能鉴定出明确的代谢产物，但本研究将研究重点聚焦于原型成分，与原型成分相比，代谢产物存在结构未知多样、浓度极低、化学性质差异大等特点，同时，代谢物在样本采集、处理、储存过程中更易降解或变化^［22］^。而原型成分结构已知，稳定性高，质谱相应稳定^［23］^。该研究在较全面化学表征的基础上，结合体内外分析，明确了玉屏风颗粒中多种活性成分的体内暴露情况，为其药效物质基础研究及质量控制奠定重要依据。
